# General Medicine and Therapeutics

**Published:** 1893-12

**Authors:** 


### *SELECTIONS AND ABSTRACTS.*

### GENERAL MEDICINE AND THERAPEUTICS.

EDITED BY DR. LUTHER B. GRANDY.

### THE PERIOD OF INCUBATION OF THE INFECTIVE FEVERS.

The period of incubation of the various infective fevers, says the *Medical Recorder* has been a matter of great discussion, and one concerning which definite conclusions have not been reached. A report on this subject by a committee of the London Clinical Society will be received with much interest.

The material which the report contains was obtained in response to a circular letter issued in 1889 by a committee over which Dr. Broadbent presided. The large mass of documents received was digested by Dr. Dawson Williams, one of the honorary secretaries of the committee. Dr. Williams was at the pains to go through the whole reports made by its medical inspectors to the Medical Department of the Local Government Board since 1878, which had been laid open to the inspection of the committee by Sir George Buchanan, one of its members. Many important facts and observations in respect especially to diphtheria, typhoid fever, and scarlet fever, were thus obtained.

The system upon which the summaries were prepared has been to take first those cases in which the exposure to the source of infection was for a short time—a few minutes or hours—at a known date. These have been made "the basis of the conclusions drawn as to the duration of incubation; while other histories, in which only the date of the commencement or cessation of exposure to a source of infection was given, has been used for contributory evidence." The duration of infectiousness is also investigated by the light of the data supplied; and the length of time which a patient who has suffered from the disease should be isolated, the period during which a susceptible person exposed to infection should be quarantined, the liability to the retention of infection in clothes and to its dissemination by milk and water, are also considered.

The conclusions of a committee which has evidently gone over the subject so carefully are of much importance, and deserve to be presented here:

Diphtheria, two to seven days; oftenest two.

Typhoid fever, eight to fourteen days; sometimes twenty-three.

Influenza, one to four days; oftenest three to four.

Measles, seven to eighteen days; oftenest fourteen.

Mumps, two to three weeks; oftenest three weeks.

Rubeola, two to three weeks.

Scarlet fever, one to seven days; oftenest two to four.

Smallpox, nine to fifteen days; oftenest twelve.

Further investigations were made with regard to the time and duration of the infective period.

Diphtheria was found to be infective during the period of incubation, attack and convalescence.

Mumps and rubeola are also infective for three or four days before the onset of the parotiditis and appearance of the rash.

The contagiousness of measles speedily disappears, and does not continue in disinfected persons for over three weeks.

Typhoid fever is infectious from the time of onset until two weeks after the fever has gone and convalescence set in.

As is well known, the contagiousness of scarlet fever varies greatly, but is generally continued a very long time—certainly until desquamation ceases, and sometimes as long as eight weeks.

The figures put forth by the committee of the Clinical Society will doubtless excite some discussion and controversy, but they are based on a large amount of clinical data.—*Clinical Reporter.*

---

### THINGS WORTH REMEMBERING.

It is authoritatively stated that headache almost always yields to the simultaneous application of hot water to the feet and back of the neck.

Ordinarily one woman in eight is sterile, but among women who have fibroids one in three is sterile. (Parvin.)

In facial erysipelas, where you cannot conveniently apply ordinary means, paint the part with ten per cent. iodoform collodion. (Prof. Gross.)

In posterior displacements of the uterus, always replace the organ before introducing a pessary ; the frequent failure of its use is generally due to this cause. (Parvin.)

Where there is a collection of foreign matter, as pus, in the antrum of Higmore, extract the first molar tooth (or more, if necessary), and drain the cavity in this way. (Sajous.)

For specific vaginitis, Prof. Parvin ordered mucilaginous injections and warm hip-baths in the acute stage, followed by injections of 1 : 100 corrosive solutions and tampons of boracic acid and glycerine.

Gelsemium will often do more good in irritable bladder than any other remedy. It is especially adapted to those women of hysterical type troubled by irritability at the neck of the bladder, calling for constant urination.

Without exception, the first symptom of pregnancy is an increased frequency of the desire to micturate.

Rhus aromatica, or the fragrant sumach, which grows all through the Northern States, is strongly recommended for incontinence of urine in atonic states of the bladder. From ten to fifteen drops of the tincture are given three times a day.

Salicylic acid is highly recommended as an application to ringworm. It may be used as an ointment, but is much better as a saturated solution in collodion. One application is often all that is necessary to effect a cure, but it may be repeated if necessary. The pain caused is not usually severe.

Boro-tartrate of potassium is the first remedy for calculus in pelvis of kidney ; a week solution must be used, and for a long time, a strong being detrimental. (Bartholow.)

Drop into urine in a test tube a few drops of the tincture of guaiac, heat it about 100°, and if it turns pale blue, pus is present in the urine.

Houghton, of Dublin, says that two honrs of severe mental labor abstracts as much vital strength from the system as a whole day of physical labor.

Unna treats "red nose" with zinc-and-sulphur oitment externally and ichthyol internally.—*Massachusetts Medical Journal. Canada Lancet.*

---

### RECTAL INJECTION OF SALINE SOLUTION IN A CASE OF SEVERE HEMORRHAGE DURING ABORTION.

I was lately called to see a woman, aged thirty, who had just aborted, the process being accompanied by very severe hemorrhage, which had nearly ceased on my arrival. The patient was the mother of three children born alive; all the labors had been accompanied by severe loss. So great had been the hemorrhage in her last confinement that her medical attendant warned her against the risk of a further increase in her family. I found the patient in a deplorable condition from the acute anemia. The pulse was 140 to 150, small, running and occasionally irregular; the cardiac sounds were faint and the apex beat imperceptible. She was extremely pallid and restless, and could not get enough air. She complained of nausea, with constant retching and vomiting of small quantities of mucus. Every few minutes she became thirsty and then faint, and was unconscious at times. Notwithstanding treatment by raising the foot of the bed, bandaging the legs, hypodermic injection of brandy, etc., the faintness, sickness and feeble pulse continued, the patient becoming even more prostrate. I determined after an hour to try the effect of a chloride of sodium enema, one drachm to the pint of water, at the temperature of 100°. The patient having had severe diarrhea, I was afraid the injection would not be retained, but this was successfully accomplished by holding a pad over anus for a few minutes. The effect was extremely beneficial, for within twenty minutes the pulse fell to 120, the color improved and the sickness and faintness almost entirely disappeared, and she expressed herself as feeling much better and more comfortable. In half an hour she was able to take and keep down a small quantity of beef extract. Shortly after, she had a little sleep. The patient made an excellent recovery and was able to be out in about a month later. I feel confident that the saline enema saved the patient's life.—*Dr. Nicholson, in Lancet. Clinical Reporter.*

---

### TREATMENT OF CHRONIC VALVULAR DISEASE OF THE HEART.

Dr. James Tyson, *Therap. Gaz.*, discusses the various heart tonics used in the treatment of chronic valvular disease of the heart.

Digitalis is a remedy always better intermitted to obtain its best effects. He believes that the greater apparent efficiency of the infusion is partly due to the fact that it is generally given in larger doses. One-half an ounce is not an infrequent dose of the infusion. This represents nearly three grains of the powder, or twenty minims of the tincture. Of remedies which may be substituted for digitalis, stropanthus should, perhaps, be first mentioned, not that it is always the best. Dr. Tyson has used it in doses of ten minims or twenty drops every two hours for forty-eight hours, without interruption and with good results. It is undoubtedly better borne by the stomach than digitalis. Caffeine is an admirable heart tonic in mitral regurgitation. One should not give less than three grains at a dose, seldom more, every three hours. When caffeine has been given in full doses for some time it produces mental symptoms quite characteristic, consisting in hallucinations. They, however, cease immediately when the drug is discontinued. Another effect of caffeine which sometimes interferes with its usefulness is its effect in inducing insomnia. Sparteine sulphate, the author values very highly, especially where a diuretic effect is desired. The dose to rely upon is never less than one-fourth of a grain, increased to one-half of a grain, three, four, and five times a day.—*Can. Lancet.*

---

### THE TREATMENT OF DIABETES.

Harley, *British Medical Journal*, after speaking of the remedies generally employed in diabetes, such as opium, morphine, codeine, hyoscyamus, cocaine, bromide of ammonium and the like, calls attention to the value of croton chloral. He says: "The beneficial effects of croton chloral often surprise me, but I seldom ever give it except in combination with a vegetable narcotic or anodyne. . . . To give a single formula that will answer in every case of a protean disease like diabetes is, however out of the question, so all I will do is to give the one employed in the case just alluded to. It is the following:

℞. Croton chloral, $\frac{1}{2}$ gr.

Opii, j gr.

Ext. aloes barb., $\frac{1}{6}$ gr.

Ext. gentianæ, jss gr.

M. Sig.: One pill to be taken three times a day."

In cases of diabetes resulting from nervous exhaustion, as well as those arising from pancreatic disease, he states that he found extract of nux vomica in one-fourth grain doses three times a day in a croton chloral pill of great service. In such cases remedies tending to improve the general health, notably phosphoric and nitro-hydrochloric acids, are of service. He has found antipyrin and peroxide of hydrogen very useful in one or two instances.—*Canada Lancet.*

---

### ANTISEPTIC CATHARTIC.

Eichler employs the following prescription as a cathartic and internal antiseptic:

R. Salol, ʒ ij.

Castor oil, ʒ vj.

Syrup of rhubarb, ℥ iss.

Cinnamon water, ℥ v.

Powder gum arabic, q.s.

Make into an emulsion, and administer one tablespoonful every hour until a purgative effect is obtained, in cases of chronic diarrhea; or else one full dose may be employed, using at the same time a disinfectant rectal imjection, containing fifteen grains of salicylic acid to a pint of water. The diet should be composed principally of milk and beef tea.—*Charlotte Medical Journal.*

